# A Novel Adaptive Two-Stage Information Filter Approach for Deep-Sea USBL/DVL Integrated Navigation

**DOI:** 10.3390/s20216029

**Published:** 2020-10-23

**Authors:** Kaifei He, Huimin Liu, Zhenjie Wang

**Affiliations:** 1 College of Oceanography and Spaces Informatics, China University of Petroleum (East China), Qingdao 266580, China; kfhe@upc.edu.cn (K.H.); sdwzj@upc.edu.cn (Z.W.); 2 Laboratory for Marine Mineral Resources, Qingdao National Laboratory for Marine Science and Technology, Qingdao 266071, China; 3 China Geological Survey Qingdao Institute of Marine Geology, Qingdao 266071, China

**Keywords:** ultra-short baseline system, Doppler velocity log, adaptive two-stage information filter, currents velocity, measurement noise covariance

## Abstract

An accurate observation model and statistical model are critical in underwater integrated navigation. However, it is often the case that the statistical characteristics of noise are unknown through the ultra-short baseline (USBL) system/Doppler velocity log (DVL) integrated navigation in the deep-sea. Additionally, the velocity of underwater vehicles relative to the bottom of the sea or the currents is commonly provided by the DVL, and an adaptive filtering solution is needed to correctly estimate the velocity with unknown currents. This paper focuses on the estimation of unknown currents and measurement noise covariance for an underwater vehicle based on the USBL, DVL, and a pressure gauge (PG), and proposes a novel unbiased adaptive two-stage information filter (ATSIF) for the underwater vehicle (UV) with an unknown time-varying currents velocity. In the proposed algorithm, the adaptive filter is decomposed into a standard information filter and an unknown currents velocity information filter with interconnections, and the time-varying unknown ocean currents and measurement noise covariance are estimated. The simulation and experimental results illustrate that the proposed algorithm can make full use of high-precision observation information and has better robustness and navigation accuracy to deal with time-varying currents and measurement outliers than existing state-of-the-art algorithms.

## 1. Introduction

The underwater vehicle (UV) remains the best option for manipulation tasks, such as sampling, detailed inspection, and servicing subsea instruments. In deep-sea, the UV is dependent on the navigation ability to perform long-range and long-term missions near the sea bottom to support a wide range of ocean surveys. For the past decades, numerous underwater navigation schemes have been proposed [1,2,3]. Here, the ultrashort baseline (USBL) system/Doppler velocity log (DVL) integrated navigation is one of the most important ones, since it provides absolute position and is not prone to dead reckoning error in deep-sea. In particular, the USBL system and the DVL are complementary, and conveniently installed in the hull and extensively applied to underwater positioning [4,5]. Additionally, the UV is usually outfitted with a pressure sensor, and the vehicle depth is computed from the direct measurements of the ambient sea water pressure via standard equations for the properties of sea water [6,7]. Combined with the calibration and compensation for depth information, the three-dimensional navigation of the underwater vehicle can commonly be converted into two-dimensional navigation.

The DVL, by the principle of Doppler frequency shift, can be used to calculate the velocity of the vehicle relative to the sea bottom or the water column, and obtain a displacement by velocity integration [8,9,10]. In the mid-depth zone, the vehicle velocity measured by the DVL can be influenced by ocean currents. Acoustic navigation systems are the only effective sensors for horizontal state measurements in the mid-depth zone. Since the current velocity is typically unknown, estimating the correct vehicle and current velocity may have great uncertainties. Additionally, the acoustic navigation methods, including the USBL, require accurate alignment calibration and sound velocity profiles (SVPs). The measurement accuracy of the USBL is influenced by the range error and bearing error, which decreases according to the increase of the distance between the transponder and transducer. As a result, the observation noise of the USBL varies violently with observation distance, and it shows poor positioning accuracy without the aid of other sensors in the deep-sea. When the elevation of real-time average sea level is known, it is convenient to combine the USBL system with a pressure sensor for navigation near the seabed. The slant range measurements can be adopted to improve the horizontal positioning accuracy [11]. Since they are unknown, saltation varying current disturbances are more difficult to compensate by using integral effect or adaptation, and the convergence of traditional navigation method can be relatively slow [12].

Reliable state/parameter estimation is a prerequisite for the stability and performance of navigation systems. The state estimation under arbitrary unknown inputs has received much attention in the past decades. As extensions of traditional Kalman filtering, augmented Kalman filters (AKFs) are frequently applied to the joint estimation of linear motion with colored noise or unknown input, and related work can be found in types of research [13,14,15,16,17]. Friedland proposed a classical two-stage Kalman filter, which decomposes the augmented filter into two reduced-order filters [14]. However, the approach is only optimal in the presence of a constant bias [15]. Kitanidis first developed an unbiased recursive filter without prior information about the unknown input [16]. However, the limiting condition of the approach is it requires the assumption that the distribution matrix of the unknown inputs in the measurement equation is of full rank. A global optimal filter was proposed, which removed this assumption, but this filter is limited to state estimation presenting a unified approach to design a specific globally optimal state estimator [17]. Recently, particle filters have also been applied to unknown input estimation, and they can cope with systems with non-Gaussian noise [18,19,20]. Other relevant examples include robotics or advanced vehicle applications where the applied forces or acceleration are unmeasured and can change arbitrarily due to the environment [21,22,23,24,25,26,27,28].

Due to the time-variant measurements noise and unknown ocean currents, an adaptive filtering solution is required to estimate the position of the UV. In practice, it is not natural to assume directly the unknown input properties and mathematical model of the augmented system in a complicated marine environment. The strong time-varying characteristics of the colored noise or unknown input, for example, the saltation unknown ocean currents, should be considered. Another disadvantage of AKF is that it requires an accurate statistical model [29], which is difficult and even impractical in underwater acoustic application. As above, the measurement accuracy of USBL observation is affected by the environment and varies dramatically, which is not conducive to the stability of filtering. Without precise models/properties for the unmolded dynamics, accurate estimation is still necessary for underwater vehicle monitoring purposes. The covariance matrix of the observation equation can be approximately estimated by the most common adaptive Sage filtering, according to window smoothing of the innovation sequence or residual sequence, which is called innovation-based adaptive estimation (IAE) and residual-based adaptive estimation (RAE) [30,31,32]. The process noise covariance matrix ***Q*** or the measurement noise covariance matrix ***R*** with time can be estimated by the IAE and RAE algorithms. When the system is subject to an abnormal condition, an adaptive factor is utilized by the robust adaptive Kalman filter (RAKF) proposed by Yang and Gao [33] to tune the predicted state error covariance. However, the IAE, RAE, and RAKF methods are all described based on the information provided by the innovation or the residual. Under the effect of unknown ocean currents, the estimation of measurement noise covariance can be damaged, and thereby lead to inaccurate and even divergent output of the filter.

In this study, a new filter is proposed to estimate the unknown time-varying currents and the real-time observation covariance matrix. The main contribution is the design of the unbiased adaptive two-stage information filters (ATSIFs) for deep-sea underwater vehicles to estimate the position, velocity, and time-varying unknown current velocity. The ATSIF involves two interconnected parts, one based on the classical information filter for state estimation, and the other based on the sequential least squares algorithm for unknown parameter estimation. The different forgetting factors are introduced to classical sequential least squares, and it controls how fast past observations are forgotten. An adaptive estimation method of the measurement noise is presented based on the epoch difference in the measurements. The acoustic system error caused by inaccurate sound velocity can be estimated by the ATSIF as well, which is convenient to be applied in our underwater navigation research. In addition, measurement equations for the USBL, DVL, and PG sensors are given, and simulation and experimental results are presented.

This paper is arranged as follows. Section 2 introduces the navigation system for the USBL/DVL integrated navigation system based on the slant ranges and the depth measurements. Then, the dynamic and measurement equations, and the data integration equations are given. The unbiased adaptive two-step information filter is described in Section 3. Section 4 describes the simulation and experiments. Finally, Section 5 presents the conclusions of this study.

## 2. Integrated Navigation Model

### 2.1. System Model

**Figure 1:**
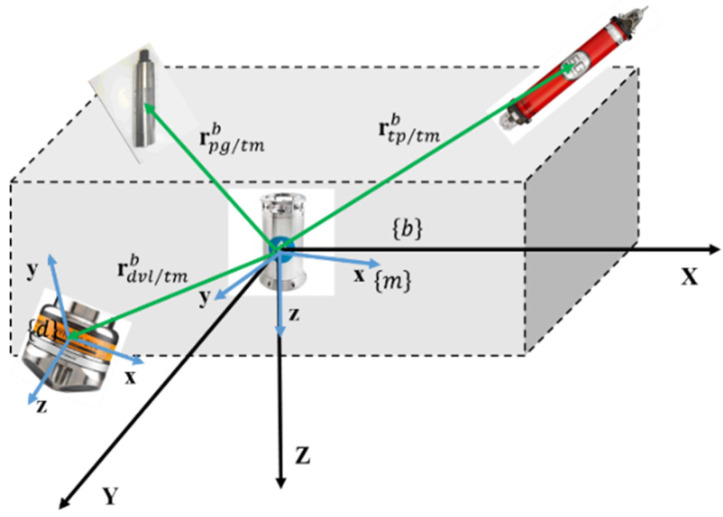
Relative positions of sensors on the underwater vehicles.

As shown in Figure 1, the vehicle fixed coordinate system {**b**} and the outline of the vehicle are simplified as a dashed box. The positions of the sensor packages relative to the attitude and heading reference system (AHRS) are given by the lever arms vectors $r_{\mathrm{tp}/\mathrm{tm}}^{b}$,$r_{\mathrm{dvl}/\mathrm{tm}}^{b}$,$r_{\mathrm{pg}/\mathrm{tm}}^{b}$ for the USBL transponder, DVL, and pressure gauge in the vehicle fixed frame, respectively. 

The positions of the other sensors relative to the AHRS in the {**m**} coordinate are represented as $r_{\mathrm{tp}}^{m}$, $r_{\mathrm{dvl}}^{m}$, and $r_{\mathrm{pg}}^{m}$, respectively. Then [34]:

(1) $$r_{\mathrm{tp}}^{m}=C_{b}^{m}(\varphi_{\mathrm{bm}})r_{\mathrm{tp}/\mathrm{tm}}^{b},\;r_{\mathrm{dvl}}^{m}=C_{b}^{m}(\varphi_{\mathrm{bm}})r_{\mathrm{dvl}/\mathrm{tm}}^{b},\;r_{\mathrm{pg}}^{m}=C_{b}^{m}(\varphi_{\mathrm{bm}})r_{\mathrm{pg}/\mathrm{tm}}^{b},$$

where $C_{b}^{m}(\varphi_{\mathrm{bm}})\in {\mathbb{R}}^{3\times 3}$ denotes the direction cosine matrix (DCM) from {**b**} to {**m**}, $\varphi_{\mathrm{bm}}$ is the attitude angle of {**b**} relative to {**m**}.

The dynamics of the position and attitude are given by:

(2) $${\dot{p}}_{m}^{n}(t)=C_{m}^{n}(\varphi_{\mathrm{mn}}(t))v_{d}^{m}(t)+v_{c}^{n}(t)+w_{v}(t),$$

(3) $${\dot{C}}_{m}^{n}(\varphi_{\mathrm{mn}}(t))=C_{m}^{n}(\varphi_{\mathrm{mn}}(t))S(\omega_{m}^{m}(t))+w_{\varphi }(t),$$

where $p_{m}^{n}(t)\in {\mathbb{R}}^{3}$ is the position of AHRS in the local navigation coordinate frame {**n**}; $C_{m}^{n}(\varphi_{\mathrm{mn}}(t))\in {\mathbb{R}}^{3\times 3}$ denotes the DCM from {**m**} to {**n**}, and $\varphi_{\mathrm{mn}}(t)$ represents the attitude of the AHRS; $v_{d}^{m}(t)\in {\mathbb{R}}^{3}$ and $\omega_{m}^{m}(t)$ represent the velocity of the AHRS relative to the fluid and angular velocity in {**m**}, respectively; $v_{c}^{n}(t)$ denotes the ocean currents velocity in {**n**}; S(⋅) is the skew-symmetric matrix, which represents the cross product such that $S(\omega_{m}^{m}(t))a=\omega_{m}^{m}(t)\times a$; $w_{v}(t)\in {\mathbb{R}}^{3}$ and $w_{\varphi }(t)\in {\mathbb{R}}^{3}$ represent the state stochastic perturbations of the velocity and turn rate, respectively.

### 2.2. Observation Model

**Figure 2:**
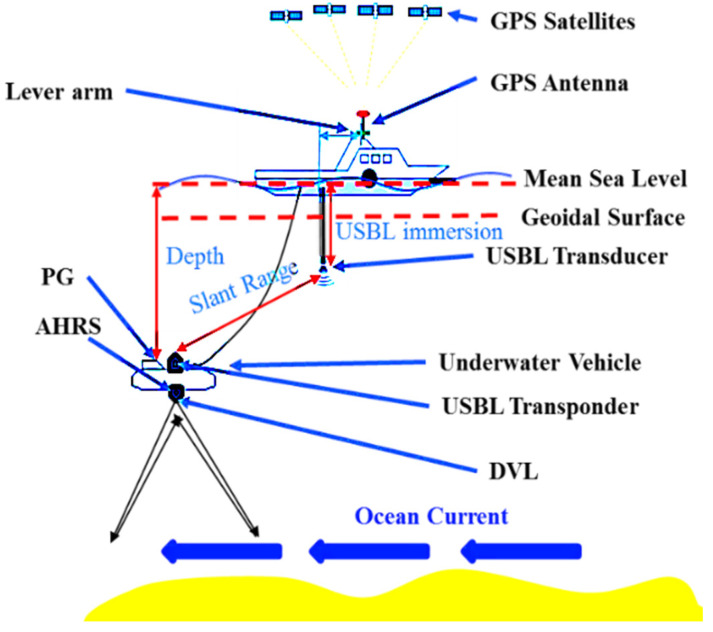
Deployment and basic functionality of the ultra-short baseline (USBL) system and the geometric relationship between the global positioning system (GPS), doppler velocity log (DVL), attitude and heading reference system (AHRS), and pressure gauge (PG) sensors.

As shown in Figure 2, the navigation system includes two parts: (1) An underwater vehicle equipped with a DVL sensor, PG, AHRS, and USBL transponder; and (2) a ship-decked unit with a GNSS antenna and a dunking transducer on a rigid pole. The relative coordinates in the {**n**} frame of the GNSS antenna and the USBL transducer can be calculated by attitude measurements. The relative position between the vehicle and ship can be measured by USBL, and the UV depth relative to the sea level can be communicated with the ship using an acoustic modem.

The measurement equations for the USBL transponder [8,35,36,37] and the DVL [26] are given by Equations (4) and (5), respectively:

(4) $$d_{i}(t)={\left\|p_{m}^{n}(t)+C_{m}^{n}(\varphi_{\mathrm{mn}}(t))r_{\mathrm{tp}}^{m}-p_{r}^{n}(i)\right\|}_{2}+\varepsilon_{i}^{\mathrm{tp}}(t)\begin{array}{cc} , & i=1,\ldots ,n_{r} \end{array},$$

(5) $$v_{\mathrm{dvl}}^{d}(t)=C_{m}^{d}(\varphi_{md})(v_{m}^{m}(t)+\omega_{m}^{m}(t)\times r_{\mathrm{dvl}}^{m}(t))+\varepsilon_{\mathrm{dvl}}^{d}(t),$$

where $d_{i}(t)$ denotes the distance between the transponder and the transducer measured by the USBL; $p_{r}^{n}(i)\in {\mathbb{R}}^{3}$ is the position of the receiver in {**n**}, and $n_{r}$ is the number of receivers; $\varepsilon_{i}^{\mathrm{tp}}(t)$ represents the USBL measurement noise; $v_{\mathrm{dvl}}^{d}(t)\in {\mathbb{R}}^{3}$ represents the velocity reading provided by the DVL; $C_{m}^{d}(\varphi_{\mathrm{md}})$ is the rotation matrix from {**m**} to {**d**}; $\varepsilon_{\mathrm{dvl}}^{d}(t)$ represents the DVL measurement noise.

In this research, the positioning accuracy of the vertical direction is better than 30 cm, which uses the differential global positioning system (DGPS) technology (e.g., StarFire, VeriPos, and Marinestar) based on the communication data link of marine satellites [38]. Then, the instantaneous elevation of the sea surface z(t) can be simplified, as represented by:

(6) $$z(t)=z_{\mathrm{tp}}(t)-z_{d}+w_{\mathrm{tp}}^{d}(t),$$

where $z_{\mathrm{tp}}(t)$ denotes the height of the real-time USBL transducer; $z_{d}$ represents the immersion of the transducer, which is usually assumed to be a constant and can be estimated; and $w_{\mathrm{tp}}^{d}$ represents the noise caused by the waves and the ship dynamic draft. Typically, the period of a wave is only a few seconds to a few minutes, while the cycle of tides is longer and can be considered almost constant within several minutes. The influence of waves can be removed by roughly utilizing the moving average method. Therefore, considering that the PG is calibrated, the measurement $p_{\mathrm{pg}}(t)$ can be represented by:

(7) $$p_{\mathrm{pg}}^{n}(t)=p_{\mathrm{atm}}(t)+\rho (t)gz_{\mathrm{pg}}^{n}(t)+\varepsilon_{\mathrm{pg}}^{}\;\mathrm{and}\;z_{\mathrm{pg}}^{n}(t)=z_{m}^{n}(t)+[\begin{array}{ccc} 0 & 0 & 1 \end{array}]C_{m}^{n}(\varphi_{\mathrm{mn}}(t))r_{\mathrm{pg}}^{m}+\varepsilon_{\mathrm{pg}}^{n},$$

where $p_{\mathrm{atm}}(t)$ denotes the atmospheric pressure at the mean sea level; ρ(t) represents the mean seawater density; g is the acceleration of gravity; $z_{\mathrm{pg}}^{n}$ denotes the depth of the pressure gauge; $\varepsilon_{\mathrm{pg}}^{}$ represents the PG noise; $z_{m}^{n}(t)$ represents the depth of the AHRS; and $\varepsilon_{\mathrm{pg}}^{n}$ represents the depth offset error.

### 2.3. Integrated Navigation Model

To reduce the complexity of the system dynamics and measurements, the calibration was accomplished and the exact orientation vector was obtained. A simple linear time-varying (LTV) model is used here:

(8) $$\dot{x}(t)=A(t)x(t)+B(t)s(t)+d(t),$$

where $x={[(p_{m}^{n}{)}^{T},\;(v_{m}^{m}{)}^{T}]}^{T}$, and $s=v_{c}^{n}$; $d_{k}$ and $v_{k}$ represent the vector of white Gaussian acceleration noises with zero mean, respectively; $A_{k}=\left[\begin{array}{cc} 0_{3\times 3} & C_{m}^{n}(\varphi_{\mathrm{mn}}(k))\\ 0_{3\times 3} & 0_{3\times 3} \end{array}\right]\in {\mathbb{R}}^{6\times 6}$, $B_{k}={\left[\begin{array}{cc} I_{3\times 3} & 0_{3\times 3} \end{array}\right]}^{T}$. To achieve a discrete-time model of the dynamics (4), we assumed that the measurements are obtained with a constant sampling rate *T*. Then, the system can be described by:

(9) $$\left\{ \begin{array}{l} x_{k}=\Phi_{k}x_{k-1}+\Gamma_{k}u_{k-1}+w_{k}\\ \begin{array}{cc} y_{k}=H_{k}x_{k}+v_{k} & \end{array} \end{array}\right.,$$

where $\Phi_{k}=e^{\int_{0}^{T}A(T-\tau )d\tau }$, $\Gamma_{k}={Ι}_{6}$, and $u_{k}=\int_{0}^{T}e^{A(T-\tau )}B(T-\tau )s(T-\tau )d\tau$. $H_{k}$ can be linearized and described by (4), (5), and (7) with the known parameters {$\varphi_{\mathrm{mn}}(t)$, $\varphi_{\mathrm{md}}$, $r_{\mathrm{tp}}^{m}$, $p_{r}^{n}(i)$, $r_{\mathrm{dvl}}^{m}(t)$, $z_{tp}(t)$, $r_{\mathrm{pg}}^{m}$}, and observations $y_{k}={\left[\begin{array}{ccccc} d_{1}(k) & \cdots & d_{n_{r}}(k) & {\left(v_{\mathrm{dvl}}^{d}(k)\right)}^{T} & z_{\mathrm{pg}}^{n}(t) \end{array}\right]}^{T}$. This is a simplified from for the design of an observer, as both the input and output of the system are known continuous bounded signals. Then, the classic augmented discrete-time model is usually used as follows:

(10) $$\{\begin{array}{c} \left[\begin{array}{c} x_{k+1}\\ u_{k+1} \end{array}\right]=\left[\begin{array}{cc} \Phi_{k+1} & \Gamma_{k+1}\\ 0 & I_{3} \end{array}\right]\left[\begin{array}{c} x_{k}\\ u_{k} \end{array}\right]+\left[\begin{array}{c} w_{k}\\ \xi_{k} \end{array}\right]\\ y_{k}=[\begin{array}{cc} H_{k} & 0 \end{array}]\left[\begin{array}{c} x_{k}\\ u_{k} \end{array}\right]+v_{k} \end{array}$$

where $\xi_{k}$ represents the random error. Thus, the KF method can be used for data integration in the system (10). However, the augmented form generally simplifies the physical model of the input error and neglects the strong time-varying characteristics of the unknown input $u_{k}$.

As shown in Table 1, we analyzed the influence of the unknown currents error on the system in this research and separated the unknown input from the augmented filter. The problem addressed in this study and the measurement characteristics of sensors can be summarized in the following statement.

1. The USBL observation contains obvious measurement noise, which is related to the distance and changes with space and time.
2. The other systematic errors, such as calibration errors and constant deviations of depth gauges, can be corrected by augmenting parameters.
3. There are time-varying and saltation ocean currents.

## 3. Adaptive Two-Stage Information Filter Design

To improve the estimation accuracy of the navigation state, unknown ocean current velocity, and measurement noise covariance, a modified two-stage adaptive information filter is proposed to trade the algorithm simplicity and efficiency.

### 3.1. The Two-Stage Information Filter

Considering a stochastic linear discrete-time system (10), $w_{k}$ and $v_{k}$ are independent random noise vectors with covariance matrices $Q_{k}=E[w_{k}w_{k}^{T}]$ and $R_{k}=E[v_{k}v_{k}^{T}]$, respectively. If the state estimation ${\hat{x}}_{k-1}^{}$, $P_{k-1}^{}$, and unknown parameter estimation ${\hat{u}}_{k-1}$ are given, according to system (10) and the variance-covariance propagation law, the predicted state and covariance are given as:

(11) $${\hat{x}}_{k|k-1}^{}=\Phi_{k}{\hat{x}}_{k-1}^{}+\Gamma_{k}{\hat{u}}_{k-1},\;P_{k|k-1}^{}=\Phi_{k}P_{k-1}^{}\Phi_{k}^{T}+Q_{k}.$$

The following information filter estimation can be obtained [39,40]:

(12) $${\hat{x}}_{k}^{}={\left(H_{k}^{T}R_{k}^{-1}H_{k}+P_{k|k-1}^{-1}\right)}^{-1}(H_{k}^{T}R_{k}^{-1}y_{k}+P_{k|k-1}^{-1}{\hat{x}}_{k|k-1}^{})≜N_{k}^{-1}(H_{k}^{T}R_{k}^{-1}y_{k}+P_{k|k-1}^{-1}{\hat{x}}_{k|k-1}^{}),$$

where $N_{k}^{}=(H_{k}^{T}R_{k}^{-1}H_{k}+P_{k|k-1}^{-1})$. The ${\hat{x}}_{k|k-1}^{}$ in Equation (11) corresponds to ${\hat{x}}_{k-1}^{}$ and ${\hat{u}}_{k-1}$, respectively. The ${\hat{x}}_{k}^{}$ can be determined by the two kinds of parameters based on Equation (8) as well. Therefore, ${\hat{x}}_{k}^{}$ can be written as into ${\hat{x}}_{k}^{}={\hat{x}}_{k}^{a}+{\hat{x}}_{k}^{u}$ with:

(13) $${\hat{x}}_{k}^{a}=N_{k}^{-1}(H_{k}^{T}R_{k}^{-1}y_{k}+P_{k|k-1}^{-1}\Phi_{k}{\hat{x}}_{k-1}^{a}),$$

(14) $${\hat{x}}_{k}^{u}=N_{k}^{-1}(P_{k|k-1}^{-1}\Phi_{k}{\hat{x}}_{k-1}^{u}+P_{k|k-1}^{-1}\Gamma_{k}{\hat{u}}_{k-1}),$$

where the Equation (13) is the classic information filtering equation of the system (10) without considering the unknown input ${\hat{u}}_{k-1}$, which can be calculated recursively in the form of standard information filtering. Considering the effect of Gaussian noise, and u change over time, the filtering estimates of Equation (14) can be written as:

(15) $${\hat{x}}_{k}^{u}=N_{k}^{-1}(P_{k|k-1}^{-1}\Phi_{k}{\hat{x}}_{k-1}^{u}+P_{k|k-1}^{-1}\Gamma_{k}{\hat{u}}_{k-1})+\Delta_{k-1},$$

where $\hat{u}$ represents an estimation of u, and $\Delta_{k-1}$ is the compensation term of the estimation of system noise ${\hat{u}}_{k-1}$.

It is assumed that a time-varying matrix $F_{k}\in R^{n\times p}$ exists, such that ${\hat{x}}_{k}^{u}=F_{k}{\hat{u}}_{k}$, and holds for all time. The detailed description of the similar methods can be found in [41,42]. Then:

(16) $$F_{k}{\hat{u}}_{k}=N_{k}^{-1}(P_{k|k-1}^{-1}\Phi_{k}F_{k-1}{\hat{u}}_{k-1}+P_{k|k-1}^{-1}\Gamma_{k}{\hat{u}}_{k-1})+\Delta_{k-1},$$

where the recursive linear equation of state estimation ${\hat{x}}_{k}^{u}$ is established based on $\hat{u}$. The estimation ${\hat{u}}_{k}$ of the system noise can be represented as ${\hat{u}}_{k}={\hat{u}}_{k-1}+\Delta {\hat{u}}_{k}$. Then:

(17) $$F_{k}({\hat{u}}_{k-1}+\Delta {\hat{u}}_{k})=N_{k}^{-1}(P_{k|k-1}^{-1}\Phi_{k}F_{k-1}{\hat{u}}_{k-1}+P_{k|k-1}^{-1}F_{k}{\hat{u}}_{k-1})+\Delta_{k-1}.$$

Letting $\Delta_{k-1}=F_{k}\Delta {\hat{u}}_{k}$, then (13) becomes:

(18) $$F_{k}{\hat{u}}_{k-1}=N_{k}^{-1}(P_{k|k-1}^{-1}\Phi_{k}F_{k-1}{\hat{u}}_{k-1}+P_{k|k-1}^{-1}\Gamma_{k}{\hat{u}}_{k-1}),$$

and a recursive expression of $F_{k}$ can be obtained as follows:

(19) $$F_{k}=N_{k}^{-1}(P_{k|k-1}^{-1}\Phi_{k}F_{k-1}+P_{k|k-1}^{-1}\Gamma_{k}).$$

Now, Equation (15) can be rewritten as:

(20) $${\hat{x}}_{k}^{u}=N_{k}^{-1}(P_{k|k-1}^{-1}\Phi_{k}{\hat{x}}_{k-1}^{u}+P_{k|k-1}^{-1}\Gamma_{k}{\hat{u}}_{k-1})+F_{k}({\hat{u}}_{k}-{\hat{u}}_{k-1}).$$

Note that the right second term of (20) is used to compensate the error caused by ${\hat{u}}_{k-1}\neq u$, and its continuous time KF counterpart can be find in [40]. Now, ${\hat{x}}_{k}^{}$ can be computed with Equations (13) and (20), then:

(21) $$\left\{ \begin{array}{l} {\hat{x}}_{k}^{}={\hat{x}}_{k}^{a}+{\hat{x}}_{k}^{u}\\ P_{k}^{}=N_{k}^{-1} \end{array}\right.,$$

where Equation (17) is the expression of the adaptive filter designed in this study, and it is necessary to consider the recursive computing problem.

The ${\hat{u}}_{k}$ is estimated with the observed values and the predicted values of the model. The innovation vector is defined as follows:

(22) $$V_{k}=y_{k}-H_{k}(\Phi_{k}{\hat{x}}_{k-1}^{a}).$$

It is straightforward to find that $V_{k}$ represents the standard Kalman filter innovation vector of the system (10) without considering $u_{k}$. Then, the covariance of $V_{k}$ can be computed by $\Sigma_{k}=R_{k}^{}+H_{k}P_{k|k-1}^{}H_{k}^{T}$.

Considering the equation ${\hat{x}}_{k-1}^{u}=F_{k-1}{\hat{u}}_{k-1}$, the innovation vector also satisfies:

(23) $$V_{k}=H_{k}\Phi_{k}F_{k-1}{\hat{u}}_{k-1}+H_{k}\Gamma_{k}{\hat{u}}_{k-1}=(H_{k}\Phi_{k}F_{k-1}+H_{k}\Gamma_{k}){\hat{u}}_{k-1}.$$

Clearly, the (23) right term can be rewritten as $H_{k}(\Phi_{k}{\hat{x}}_{k-1}^{u}+\Gamma_{k}{\hat{u}}_{k-1})$. Now, Equations (18) and (19) is subtracted with the left and right. Then:

(24) $$0=y_{k}-H_{k}(\Phi_{k}{\hat{x}}_{k-1}^{}+\Gamma_{k}{\hat{u}}_{k-1}).$$

So, the relationship between $V_{k}$ and ${\hat{u}}_{k-1}$ is given by (19) as:

(25) $$V_{k}=D_{k}u_{k-1},$$

where $D_{k}=(H_{k}\Phi_{k}F_{k-1}+H_{k}\Gamma_{k})$.

Equation (21) can be regarded as an observation equation with unknown parameters u. Based on the steady characteristics of unknown input, the sequential least squares can be used to estimate ${\hat{u}}_{k}$ sequentially. The detailed equations are given as:

(26) $$\Psi_{k}={\left(\Sigma_{k}+D_{k}\Lambda_{k-1}^{}D_{k}^{T}\right)}^{-1},$$

(27) $$G_{k}=\Lambda_{k-1}^{}D_{k}^{T}\Psi_{k},$$

(28) $$\Lambda_{k}^{}=\Lambda_{k-1}^{}-\Lambda_{k-1}^{}D_{k}^{T}\Psi_{k}D_{k}^{}\Lambda_{k-1}^{},$$

(29) $${\hat{u}}_{k}={\hat{u}}_{k-1}+G_{k}(V_{k}-D_{k}{\hat{u}}_{k-1}).$$

In the adaptive information filter, at the initial time instant *k* = 0, the initial state ${\hat{x}}_{0}\in N({\bar{x}}_{0},P_{0})$ is assumed to be a Gaussian random vector, and the basic algorithm of two-stage information filtering (TSIF) can be given as Algorithm 1.
**Algorithm 1:** Two-Stage Information Filter (TSIF).1. **Initialization:**    ${\hat{x}}_{0}^{a}∼\mathbb{N}(x_{0},P_{0})$,${\hat{x}}_{0}^{u}=0$,$F_{0}=0$,$\Lambda_{0}^{u}=\omega^{u}I_{p}$,${\hat{u}}_{0}^{}=0$.
 2. **Input:** observation $y_{k}$(30)3. **Recursive computation:** For *k* = 1, 2, 3, … (1). **Information filtering (IF):**    ${\hat{x}}_{k|k-1}^{a}=\Phi_{k}{\hat{x}}_{k-1}^{a}$,(31)    $P_{k|k-1}^{}=\Phi_{k}P_{k-1}^{}\Phi_{k}^{T}+Q_{k}$,(32)    $N_{k}^{}=(H_{k}^{T}R_{k}^{-1}H_{k}+P_{k|k-1}^{-1})$,(33)    ${\hat{x}}_{k}^{a}=N_{k}^{-1}(H_{k}^{T}R_{k}^{-1}y_{k}+P_{k|k-1}^{-1}\Phi_{k}{\hat{x}}_{k-1}^{a})$,(34)    $P_{k}^{}=N_{k}^{-1}$.(35) (2). **Innovation and covariance:**    $V_{k}=y_{k}-H_{k}(\Phi_{k}{\hat{x}}_{k-1}^{a})$,(36)    $\Sigma_{k}=R_{k}^{}+H_{k}P_{k|k-1}^{}H_{k}^{T}$.(37) (3). **Correction:**    $F_{k}=N_{k}^{-1}(P_{k|k-1}^{-1}\Phi_{k}F_{k-1}+P_{k|k-1}^{-1}\Gamma_{k})$,(38)    $D_{k}=(H_{k}\Phi_{k}F_{k-1}+H_{k}\Gamma_{k})$,(39)    $\Psi_{k}={\left(\Sigma_{k}+D_{k}\Lambda_{k-1}^{}D_{k}^{T}\right)}^{-1}$,(40)    $G_{k}=\Lambda_{k-1}^{}D_{k}^{T}\Psi_{k}$, (41)    $\Lambda_{k}^{}=\Lambda_{k-1}^{}-\Lambda_{k-1}^{}D_{k}^{T}\Psi_{k}D_{k}^{}\Lambda_{k-1}^{}$,(42)    ${\hat{u}}_{k}={\hat{u}}_{k-1}+G_{k}(V_{k}-D_{k}{\hat{u}}_{k-1})$,(43)    ${\hat{x}}_{k}^{u}=N_{k}^{-1}(P_{k|k-1}^{-1}\Phi_{k}{\hat{x}}_{k-1}^{u}+P_{k|k-1}^{-1}\Gamma_{k}{\hat{u}}_{k-1})+F_{k}({\hat{u}}_{k}-{\hat{u}}_{k-1})$.(44) (4). **Modified state:**    ${\hat{x}}_{k}^{}={\hat{x}}_{k}^{a}+{\hat{x}}_{k}^{u}$.(45)4. **Output:**
${\hat{x}}_{k}^{}$ and ${\hat{u}}_{k}$

**Proposition** **1.** *The mathematical expectations*$Ε[x_{k}^{}-{\hat{x}}_{k}^{}]\;$*and*$Ε[u_{k}-{\hat{u}}_{k}]$*tend to zero when*k→∞*. The details can be found inAppendix A*.

The Algorithm 1 separates unknown ocean currents and navigation parameters into two parts. It is more convenient to reveal the effect of unknown ocean currents parameters on the function of possible estimated parameters through the innovation vector. The ocean current parameters in different states can be estimated adaptively and more flexibly.

### 3.2. The Adaptive Estimation of Unknown Current Velocity

#### 3.2.1. Diagnosis of Unknown Ocean Currents and the Saltation Ocean Currents

When the UV works near the sea bottom, the DVL can measure the velocity relative to the bottom of the sea without ocean currents. Otherwise, many diagnostic algorithms can be applied to filter anomalies through innovation vectors, residual vectors, or abnormal states. In this study, the prediction innovation vector ${\hat{x}}_{k|k-1}^{}$ will be reflected by the error of the prediction state vector, and the following error discriminant statistics can be constructed through a sliding window of *n* epochs:

(46) $$\mathrm{mean}(\Delta e_{k}^{-})=\frac{1}{n}\sum_{j=k-n+1}^{k}\left(\frac{{\left(e_{j}^{-}\right)}^{T}e_{j}^{-}}{tr(\Sigma_{j})}\right),e_{k}^{-}=y_{k}-H_{k}(\Phi_{k}{\hat{x}}_{k|k-1}^{}).$$

The mean is compared with a presupposed threshold to distinguish whether there is an abnormal state, i.e., whether there is an unknown ocean current. If there is an unknown ocean current, the unknown parameters can be estimated by the second-order filtering.

#### 3.2.2. Diagnosis of Abnormal USBL Data

It is assumed that the location provided by USBL at *k* time is $p_{k}^{\mathrm{usbl}}$, and the predicted value of the position at the last moment is ${\hat{p}}_{k-1}^{}$. It can be obtained that the velocity at this time is estimated to be $\left(p_{k}^{\mathrm{usbl}}-{\hat{p}}_{k-1}^{})/(t_{k}-t_{k-1}\right)$. In deep sea, DVL’s speed of measurement accuracy is much higher than the average speed obtained by the adjacent time of USBL. It can be used as an important criterion to judge whether the USBL data is abnormal.

#### 3.2.3. Estimation of the Time-Varying Currents

When the time-varying ocean currents occurs, we can naturally introduce the forgetting factor λ∈[0,1] into the recursive Equations (40)–(43). Rewriting Equations (40) and (42), then the adaptive SLS algorithm for estimating the ocean current velocity is as follows:

(47) $$\{\begin{array}{l} {\bar{\Psi }}_{k}={\left(\lambda \Sigma_{k}+D_{k}{\bar{\Lambda }}_{k-1}^{}D_{k}^{T}\right)}^{-1}\\ {\bar{\Lambda }}_{k}^{}=\lambda^{-1}{\bar{\Lambda }}_{k-1}^{}-\lambda^{-1}{\bar{\Lambda }}_{k-1}^{}D_{k}^{T}{\bar{\Psi }}_{k}D_{k}^{}{\bar{\Lambda }}_{k-1}^{} \end{array}.$$

The forgetting factor is used to increase the proportion of new observations in the estimation of ocean current velocity in filtering. If there is a slow change in ocean current velocity, the method can effectively estimate the ocean currents by using new observations.

Consider the saltation ocean currents, which can cause serious effects on navigation. We consider introducing an auto-adjusting factor $\alpha_{k}$ into the above recursive Equations (40)–(43).

By rewriting Equations (40) and (42) with the adaptive two-section weight function, the SLS algorithm for estimating the ocean current velocity can be rewritten as follows:

(48) $$\{\begin{array}{l} {\tilde{\Psi }}_{k}={\left(\alpha_{k}\Sigma_{k}+D_{k}{\tilde{\Lambda }}_{k-1}^{}D_{k}^{T}\right)}^{-1}\\ {\tilde{\Lambda }}_{k}^{}=\alpha_{k}^{-1}{\tilde{\Lambda }}_{k-1}^{}-\alpha_{k}^{-1}{\tilde{\Lambda }}_{k-1}^{}D_{k}^{T}{\tilde{\Psi }}_{k}D_{k}^{}{\tilde{\Lambda }}_{k-1}^{}\\ \alpha_{k}\approx \{\begin{array}{c} 1\begin{array}{cc} & \begin{array}{ccc} & & \Delta {\tilde{v}}_{k}\leq c \end{array} \end{array}\\ \frac{c}{\Delta {\tilde{v}}_{k}}\begin{array}{cc} & \begin{array}{cc} & \Delta {\tilde{v}}_{k}\geq c \end{array} \end{array} \end{array} \end{array},$$

where $\Delta {\tilde{v}}_{k}={\left(\frac{{\left(e_{j}^{-}\right)}^{T}e_{j}^{-}}{tr(\Sigma_{j})}\right)}^{\frac{1}{2}}$ is a discriminating statistical vector based on the innovations, and c∈[1,2.5] is a presupposed threshold. The significance of Equation (48) is to reduce the influence of the previous system noise estimation on the current system noise estimation and improve the convergence of stepwise system noise. The gain matrix $\Psi_{k}$ is magnified by introducing the adaptive factor λ or $\alpha_{k}$, and depends more upon the new measurements.

The basic algorithm of adaptive two-stage information filtering (TSIF) can be given as Algorithm 2. The ATSIF involves two interconnected parts, one based on the classical information filter for state estimation, and the ther based on the sequential least squares algorithm for unknown parameter estimation. The different forgetting factors are introduced to classical sequential least squares, and it controls how fast past observations are forgotten.
**Algorithm 2:** Adaptive Two-Stage Information Filter (TSIF).1. **Initialization:**     ${\hat{x}}_{0}^{a}∼\mathbb{N}(x_{0},P_{0})$, ${\hat{x}}_{0}^{u}=0$, $F_{0}=0$, $\Lambda_{0}^{u}=\omega^{u}I_{p}$, ${\hat{u}}_{0}^{}=0$, $\lambda_{k}^{u}\in [0,1]$.(49)2. **Input: observation**
$y_{k}$
3. **Recursive computation:** For *k* = 1, 2, 3, … (1). **Information filtering (IF):**    ${\hat{x}}_{k|k-1}^{a}=\Phi_{k}{\hat{x}}_{k-1}^{a}$,(50)    $P_{k|k-1}^{}=\Phi_{k}P_{k-1}^{}\Phi_{k}^{T}+Q_{k}$,(51)    $N_{k}^{}=(H_{k}^{T}R_{k}^{-1}H_{k}+P_{k|k-1}^{-1})$,(52)    ${\hat{x}}_{k}^{a}=N_{k}^{-1}(H_{k}^{T}R_{k}^{-1}y_{k}+P_{k|k-1}^{-1}\Phi_{k}{\hat{x}}_{k-1}^{a})$,(53)    $P_{k}^{}=N_{k}^{-1}$.(54) (2). **Innovation and covariance:**    $V_{k}=y_{k}-H_{k}(\Phi_{k}{\hat{x}}_{k-1}^{a})$,(55)    $\Sigma_{k}=R_{k}^{}+H_{k}P_{k|k-1}^{}H_{k}^{T}$.(56)    $\alpha_{k}\approx \{\begin{array}{c} 1\begin{array}{cc} & \begin{array}{ccc} & & \Delta {\tilde{v}}_{k}\leq c \end{array} \end{array}\\ \frac{c}{\Delta {\tilde{v}}_{k}}\begin{array}{cc} & \begin{array}{cc} & \Delta {\tilde{v}}_{k}\geq c \end{array} \end{array} \end{array}$$,\Delta {\tilde{v}}_{k}={\left(\frac{{\left(e_{j}^{-}\right)}^{T}e_{j}^{-}}{tr(\Sigma_{j})}\right)}^{\frac{1}{2}}$, c∈[1,2.5].(57) (3). **Correction:**    $F_{k}=N_{k}^{-1}(P_{k|k-1}^{-1}\Phi_{k}F_{k-1}+P_{k|k-1}^{-1}\Gamma_{k})$,(58)    $D_{k}=(H_{k}\Phi_{k}F_{k-1}+H_{k}\Gamma_{k})$,(59)    ${\tilde{\Psi }}_{k}={\left(\alpha_{k}\Sigma_{k}+D_{k}{\tilde{\Lambda }}_{k-1}^{}D_{k}^{T}\right)}^{-1}$,(60)    ${\tilde{G}}_{k}={\tilde{\Lambda }}_{k-1}^{}D_{k}^{T}{\tilde{\Psi }}_{k}$, (61)    ${\tilde{\Lambda }}_{k}^{}=\alpha_{k}^{-1}{\tilde{\Lambda }}_{k-1}^{}-\alpha_{k}^{-1}{\tilde{\Lambda }}_{k-1}^{}D_{k}^{T}{\tilde{\Psi }}_{k}D_{k}^{}{\tilde{\Lambda }}_{k-1}^{}$,(62)    ${\hat{u}}_{k}={\hat{u}}_{k-1}+{\tilde{G}}_{k}(V_{k}-D_{k}{\hat{u}}_{k-1})$,(63)    ${\hat{x}}_{k}^{u}=N_{k}^{-1}(P_{k|k-1}^{-1}\Phi_{k}{\hat{x}}_{k-1}^{u}+P_{k|k-1}^{-1}\Gamma_{k}{\hat{u}}_{k-1})+F_{k}({\hat{u}}_{k}-{\hat{u}}_{k-1})$.(64) (4). **Modified state:**    ${\hat{x}}_{k}^{}={\hat{x}}_{k}^{a}+{\hat{x}}_{k}^{u}$.(65)4. **Output:**
${\hat{x}}_{k}^{}$ and ${\hat{u}}_{k}$


### 3.3. The Adaptive Estimation of the Measurement Noise Covariance

Due to the characteristics of acoustic sensors and ocean environment noise, the ***R*** matrix will change obviously with time and space in practice. Consequently, the performance of the filter will degrade when using an inaccurate prior statistic covariance ***R***. Therefore, it is crucial to estimate the statistical characteristics of the filter. There are numerous existing adaptive algorithms that can be used to estimate the measurement noise covariance. The IAE and RAE methods are adapted as follows:

(66) $${\hat{R}}_{k}^{\mathrm{IAE}}=E[\;e_{k}^{-}{\left(e_{k}^{-}\right)}^{T}]-H_{k}P_{k|k-1}^{}H_{k}^{T}\approx \frac{1}{m}\sum_{i=0}^{m}e_{k-i}^{-}{\left(e_{k-i}^{-}\right)}^{T}-H_{k}P_{k|k-1}^{}H_{k}^{T},$$

(67) $${\hat{R}}_{k}^{\mathrm{RAE}}=E[\;e_{k}^{+}{\left(e_{k}^{+}\right)}^{T}]+H_{k}P_{k}^{}H_{k}^{T}\approx \frac{1}{m}\sum_{i=0}^{m}e_{k-i}^{+}{\left(e_{k-i}^{+}\right)}^{T}+H_{k}P_{k}^{}H_{k}^{T},$$

where ${\hat{x}}_{k|k-1}^{}$ and ${\hat{x}}_{k}$ are the predicted and corrected estimation of the state, respectively. $P_{k|k-1}^{}$ denotes the covariance of ${\hat{x}}_{k|k-1}^{}$, and $P_{k}^{}$ denotes the covariance of ${\hat{x}}_{k}^{}$. $e_{k}^{-}$ and $e_{k}^{+}=y_{k}-C_{k}{\hat{x}}_{k}^{}$ are the innovation and residue vectors at time *k*, respectively.

However, the innovation vectors and residual vectors are sensitive to $\left({\hat{x}}_{k|k-1}^{},{\hat{u}}_{k|k-1}^{}\right)$ and $\left({\hat{x}}_{k}^{},{\hat{u}}_{k}^{}\right)$, and the inaccurate $\left({\hat{x}}_{k|k-1}^{},{\hat{u}}_{k|k-1}^{}\right)$ and $\left({\hat{x}}_{k}^{},{\hat{u}}_{k}^{}\right)$ will affect the estimation of ***R***. We consider the adaptive estimation of ***R*** from the $y_{k-1}^{p}=C_{k-1}{\hat{x}}_{k-1|k-2}^{}$ and original observations to improve the estimation accuracy. By calculating the difference of the observed data in the adjacent time, the second-order difference (SOD) of observation can be used to calculate ***R*** as follows [43]:

(68) $${\hat{R}}_{k}^{\mathrm{SOD}}\approx [Var((y_{k}-y_{k-1}^{})-(y_{k}^{p}-y_{k-1}^{p}))-H_{k}Q_{k-1}H_{k}^{T}]/2,$$

where $y_{k}^{p}=H_{k}{\hat{x}}_{k|k-2}^{}$ and the details can be found in Appendix B.

The covariance matrix of observation noise can be obtained by selecting observation data for a short period time and the moving average method. If the sampling time T is small, the observed noise covariance can be estimated by (68). The SOD method can be viewed as a second-order difference of measurement than the IAE method. In USBL/DVL integrated navigation, when the accuracy of the dynamic model is higher than that of measurement, this method can generally achieve better estimation results. The potential advantage is that it can eliminate the system error items of the observation system with little change between epochs and obtain a stable covariance matrix of observation noise, which is beneficial to parameterization and separation of measurement system errors.

**Figure 3:**
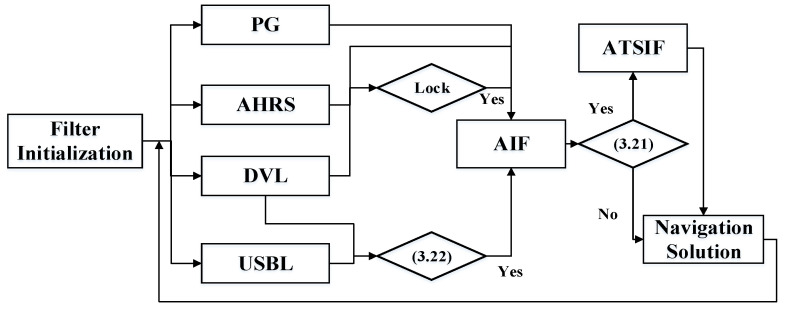
Adaptive two-stage information filter for UV navigation.

As shown in Figure 3, three discriminant conditions are referenced in the proposed filter. In order to improve the stability and accuracy of filtering, the AIF and ATSIF algorithms are used to estimate the state of the UV with different scenarios. The high-precision velocity measurement of DVL is used as the diagnostic condition of USBL anomaly observation, and the data quality of USBL can be controlled according to the equation in Section 3.2.2. When the USBL is normal and DVL is in the lock model, the AIF algorithm is used to get an accurate navigation solution. When the DVL is in the water tracking model, it is necessary to distinguish whether there are ocean currents according to the equation in Section 3.2.1. If the ocean currents occur, the ATSIF algorithm should be used to compensate the filtering error caused by the unknown currents.

## 4. Experiment Analysis

### 4.1. Simulation Results

**Figure 4:**
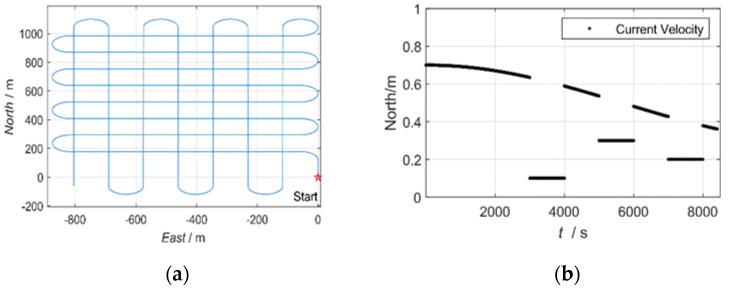
The trajectories of the underwater vehicle (**a**) and the current velocity in the northern direction (**b**).

In this section, a numerical example of an underwater vehicle tracking problem is given to illustrate the effectiveness of the proposed adaptive filtering approach. Consider an underwater vehicle with a constant velocity of 1 m/s at a depth of 1400 m. The positions of the USBL transducer and the underwater vehicle trajectory are depicted in Figure 4a, and the simulation parameters of the USBL, DVL, depth, and attitude measurements are given in Table 2.

**Figure 5:**
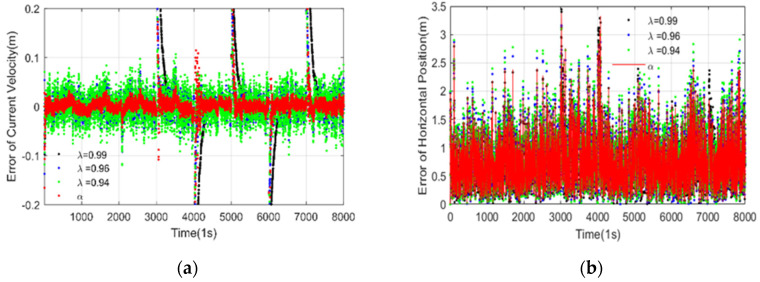
Estimated currents velocity error (**a**) and horizontal positions error (**b**). (K = 3).

The estimated current velocity error of the proposed algorithms with different adaptive factors is depicted in Figure 5a. It shows that when the adaptive factor of ***λ*** is close to 1, the historical ocean currents information occupies a large proportion in the filtering. The filtering is relatively stable at this time, but the convergence speed is slow while the saltation ocean currents happen. When the ***λ*** is small, the saltation ocean currents can be quickly estimated using the most recent observation information. However, the current estimates fluctuate greatly and the filtering shows instability. The proposed algorithm combined with the piecewise function can diagnose the time of saltation ocean currents and estimate the saltation ocean current velocity quickly and increase the stability of the ocean current estimation. The estimated horizontal position errors of the proposed algorithms are depicted in Figure 5b. It can be seen that the proposed two-section weight method gives a more accurate and robust estimation.

**Figure 6:**
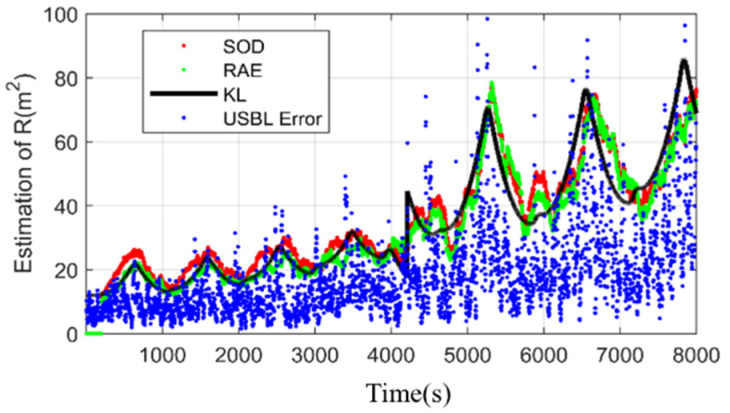
The estimation of R. (K = 5,8).

Considering the USBL accuracy changes with distance and a sudden change in the speed of sound, the scale factor K changes from 5 to 8 at 4100 epochs in this simulation. Figure 6 exhibits the estimation of USBL measurement noise covariance provided by SOD and RAE. RAE gives poor performance during the maneuvering motion because it works based on the residual sequence and is coupled with state estimation error. However, the SOD method can contribute accurate and stable estimations over almost the whole process. To validate the feasibility of the proposed algorithm, different K is implemented. Table 3 lists the root mean square errors (RMSEs) of all the approaches during different K. The proposed algorithm also outperforms other algorithms with different K.

From the simulation results, the performance of these filters is consistent with the theoretical analysis, which is stated in Section 3.

### 4.2. Deep-Sea Towed Vehicle Experiment

**Figure 7:**
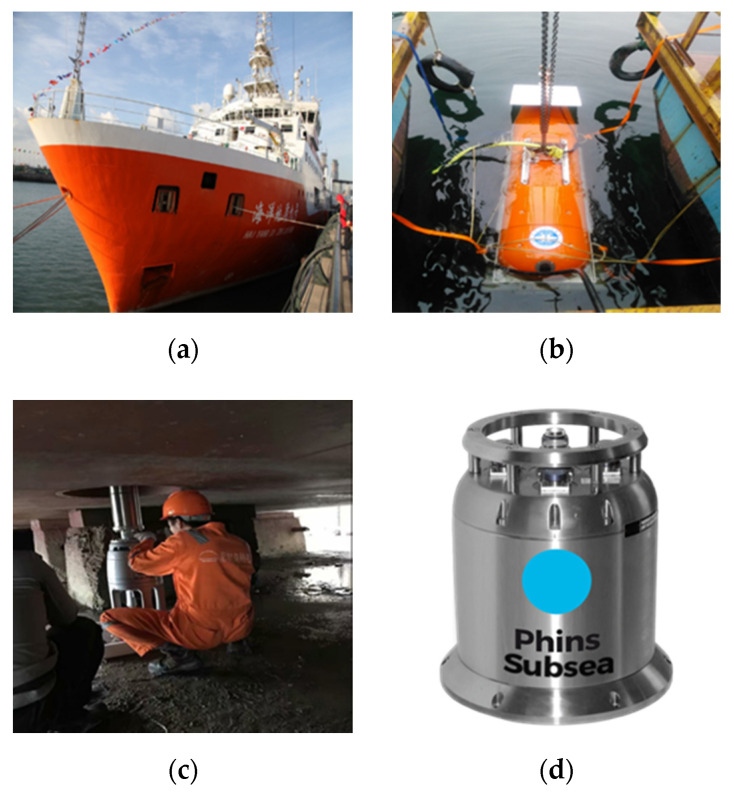
The schematic diagram of the ship (**a**), deep-sea towed vehicle (**b**), GAPS USBL (**c**), and SINS (**d**).

As shown in Figure 7, there is a deep-sea towed vehicle, GAPS ultra-short baseline acoustic positioning system, Phins Subsea strapdown inertial navigation system (SINS), and a survey vessel, respectively. The towed vehicle is equipped with an SINS, pressure gauge, DVL, and ultra-short baseline positioning system. The water depth of this experiment is about 1100 m, of which the depth meter, ultra-short baseline positioning system, and SINS inertial navigation system are the main navigation system and DVL is the auxiliary navigation system. Based on the depth data measured by the depth meter on the vertical channel, the navigation and positioning of the towing body in the horizontal direction of the deep-water area is mainly considered in this study.

**Figure 8:**
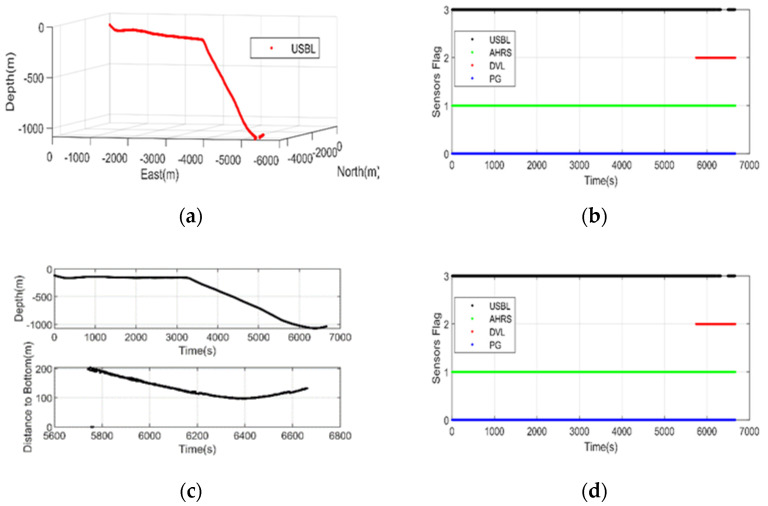
The 3-D position of USBL (**a**), usability of all navigation sensors (**b**), the PG data (**c**), and DVL velocity (**d**) in the experiment.

The DVL used in this experiment is in bottom-lock velocity measurement mode (that is, when the distance between DVL and the sea floor is less than 200 m, the velocity of the towing body relative to the seafloor can be measured without considering the influence of ocean currents). The accuracy of USBL will decrease with the increase of depth. Figure 8 shows that USBL even has the situation of missing observation data in the deep-water area (6500 s after entering the water). At this time, DVL velocity measurement information can be used as an effective supplement to restrain the navigation divergence of the SINS system to a certain extent.

Due to it being difficult to obtain the absolute positioning result of the underwater dynamic vehicle, the adaptive real-time filtering result using RAE estimation is compared and analyzed with the final post-processing navigation result obtained by the commercial post-processing software in this example. The post-processing navigation result combined with forward filtering and backward filtering with SINS data. It is considered to be a true and reliable UV position, velocity, and attitude. Since DVL does not measure the velocity relative to seawater, the measured data in this paper are only related to a part of the research content of this paper.

**Figure 9:**
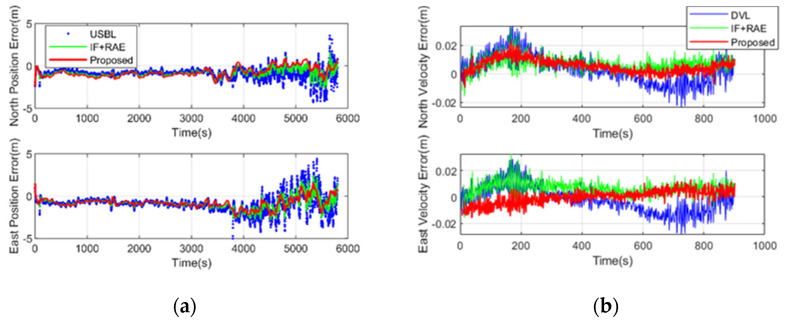
Estimated horizontal position (**a**) and velocity errors (**b**).

As shown in Figure 9, there are kinds of information filtering methods for estimating the covariance of USBL using the USBL + RAE methods and the proposed SOD adaptive filtering method. The proposed method is smoother due to the coupling of slant data of USBL and depth observations. More specifically, in the case of USBL measurements, the maximum position error is approximately 1 m in shallow water, while the maximum position error is approximately 3 m. The positions error is changed significantly with ranging. In deep water, the positioning accuracy of the USBL is significantly reduced, and real-time estimation of the USBL random model is of great significance to integrated navigation. Compared with the traditional RAE method, the proposed method obtains more stable filtering results.

## 5. Conclusions

An unbiased adaptive two-stage information filter was proposed to estimate the position of the UV and unknown current velocity. The time-varying unknown ocean current velocity and observed noise covariance were estimated, and it can be easily extended to underwater acoustic navigation filtering cases. According to simulation and experiment results, the following conclusions can be drawn: (1) The proposed approach can improve the performance of underwater acoustic navigation significantly; and (2) some stable coupling parameters, such as pressure sensors, can be effectively estimated by the new filtering algorithm. The design of the new algorithm was applied to the observation model of deep-sea acoustic navigation equipment, which provides a reference for the research of underwater integrated navigation.

## 6. Patents

In further research, the filter design with missing data of acoustic sensors needs to be considered as well. The algorithm can still be improved in the field of inertial navigation, and the adaptive estimation of system error and system noise variance in the high-dimensional navigation system are still worthy of further discussion.

## Figures and Tables

**Table 1:** Summary of measured quantities and variables to be estimated.

Estimate	Measurement
AHRS pos. $p_{m}^{n}(t)$	USBL Ranges $d_{i}(t)$
AHRS vel. $v_{m}^{n}(t)$	Relative vel. to fluid $v_{\mathrm{dvl}}^{d}(t)$
Currents vel.	Angular vel. $\omega_{m}^{m}(t)$
$v_{c}^{n}(t)$	Depth $z_{\mathrm{pg}}^{n}(t)$

**Table 2:** Simulation parameters.

	USBL (m)	DVL (m/s)	Depth (m)	Heading (°)	Roll/Pitch (°)
**Gaussian Error**	L × 0.1% × K	0.03	0.5	0.3	0.1
**System Error**	0.5 × cos (t/3600)	V × (1 + 0.05)	0.3	0.03	0.01

where L represents the slant range between the USBL transducer and the transponder; K is the scale factor; V denotes the velocity of the vehicle in the vehicle coordinates. The ocean currents direction is introduced to be north–south in this simulation, and the current velocity is simulated as Figure 4b.

**Table 3:** Average root mean square errors (RMSEs) of the UV positions with different K (m).

Method	Proposed	ARAE	IF	AIF
K = 2	1.06	1.08	1.43	1.16
K = 4	1.90	2.13	2.51	2.42
K = 8	2.47	2.79	3.20	3.17

## Nomenclature

$r_{\mathrm{tp}/\mathrm{tm}}^{b}$, $r_{\mathrm{dvl}/\mathrm{tm}}^{b}$, $r_{\mathrm{pg}/\mathrm{tm}}^{b}$	the lever arms vectors of the USBL transponder, DVL and pressure gauge
$p_{m}^{n}(t)$	the positions of AHRS in local navigation coordinate frame {**n**}
$C_{b}^{m}(\varphi_{\mathrm{bm}})$,$C_{m}^{n}(\varphi_{\mathrm{mn}}(t))$	the direction cosine matrix (DCM) from {**b**} to {**m**} and from {**m**} to {**n**}
$\varphi_{\mathrm{bm}}$	the attitude angle of {**b**} relative to {**m**} and form {**m**} to {**n**}
$\varphi_{\mathrm{mn}}$	
$d_{i}(t)$	the distance between the transponder and the transducer
$v_{d}^{m}$	the velocity of the AHRS relative to fluid and angular velocity in **{m}**
$\omega_{m}^{m}(t)$	
$v_{c}^{n}(t)$	the ocean currents velocity in {**n**}
$p_{r}^{n}$	the position of the receiver in {**n**}
$p_{\mathrm{pg}}^{n}$	the position of the pressure gauge in {**n**}
$z_{\mathrm{pg}}^{n}$	the depth of the pressure gauge in {**n**}
x	the state parameters
${\hat{x}}_{k}^{}$	the state parameters estimation

## Appendix A

Define the estimation errors are as follows:

(A1) $${\tilde{x}}_{k}^{}=x_{k}^{}-{\hat{x}}_{k}^{}\;\mathrm{and}\;{\tilde{u}}_{k}=u_{k}-{\hat{u}}_{k}.$$

According to (10) and (20), ${\tilde{x}}_{k}^{}$ can be expressed as:

(A2) $${\tilde{x}}_{k}=N_{k}^{-1}(H_{k}^{T}R_{k}^{-1}\nu_{k}+P_{k|k-1}^{-1}\Phi_{k}{\tilde{x}}_{k-1}^{}+P_{k|k-1}^{-1}\Gamma_{k}{\tilde{u}}_{k-1}+P_{k|k-1}^{-1}w_{k})+F_{k}({\tilde{u}}_{k}-{\tilde{u}}_{k-1}),$$

and according to (10) and (29), ${\tilde{u}}_{k}^{}$ can be expressed as:

(A3) $${\tilde{u}}_{k}^{}={\tilde{u}}_{k-1}^{}-G_{k}C_{k}(\Phi_{k}{\tilde{x}}_{k-1}^{}+\Gamma_{k}{\tilde{u}}_{k-1})-G_{k}C_{k}w_{k}-G_{k}\nu_{k}.$$

Define $\xi_{k}≜x_{k}^{a}-{\hat{x}}_{k}^{a}={\tilde{x}}_{k}-F_{k}{\tilde{u}}_{k}^{}$, then ${\tilde{x}}_{k-1}=\xi_{k-1}-F_{k-1}{\tilde{u}}_{k-1}$, then:

(A4) $$\begin{array}{cc} \xi_{k} & =N_{k}^{-1}(H_{k}^{T}R_{k}^{-1}\nu_{k}+P_{k|k-1}^{-1}\Phi_{k}{\tilde{x}}_{k-1}^{}+P_{k|k-1}^{-1}\Gamma_{k}{\tilde{u}}_{k-1}+P_{k|k-1}^{-1}w_{k})+F_{k}({\tilde{u}}_{k}-{\tilde{u}}_{k-1})-F_{k}\tilde{u}\\ & =N_{k}^{-1}(H_{k}^{T}R_{k}^{-1}\nu_{k}+P_{k|k-1}^{-1}\Phi_{k}{\tilde{x}}_{k-1}^{}+P_{k|k-1}^{-1}\Gamma_{k}{\tilde{u}}_{k-1}+P_{k|k-1}^{-1}w_{k})-F_{k}{\tilde{u}}_{k-1}\\ & =N_{k}^{-1}(H_{k}^{T}R_{k}^{-1}\nu_{k}+P_{k|k-1}^{-1}\Phi_{k}(\xi_{k-1}+F_{k-1}{\tilde{u}}_{k-1}^{})+P_{k|k-1}^{-1}\Gamma_{k}{\tilde{u}}_{k-1}+P_{k|k-1}^{-1}w_{k})-F_{k}{\tilde{u}}_{k-1}\\ & =N_{k}^{-1}H_{k}^{T}R_{k}^{-1}\nu_{k}+N^{-1}P_{k|k-1}^{-1}\Phi_{k}\xi_{k-1}+N^{-1}P_{k|k-1}^{-1}w_{k}+\{N^{-1}[P_{k|k-1}^{-1}\Phi_{k}F_{k-1}+P_{k|k-1}^{-1}\Gamma_{k}]-F_{k}\}{\tilde{u}}_{k-1} \end{array}$$

According $F_{k}=N_{k}^{-1}(P_{k|k-1}^{-1}\Phi_{k}F_{k-1}+P_{k|k-1}^{-1}\Gamma_{k})$, then:

(A5) $$\xi_{k}=N_{k}^{-1}H_{k}^{T}R_{k}^{-1}\nu_{k}+N_{k}^{-1}P_{k|k-1}^{-1}\Phi_{k}\xi_{k-1}+N_{k}^{-1}P_{k|k-1}^{-1}w_{k}.$$

The noises $w_{k}$ and $\nu_{k}$ are Gauss noise, then:

(A6) $$E[\xi_{k}]=N_{k}^{-1}P_{k|k-1}^{-1}\Phi_{k}E[\xi_{k-1}].$$

It is recursively shown that $E[\xi_{k}]=0$, staring from $E[\xi_{0}]=E[{\tilde{x}}_{0}]-{ϒ}_{0}E[{\tilde{u}}_{0}^{}]=0$. Similarly, substitute ${\tilde{x}}_{k-1}=\xi_{k-1}-F_{k-1}{\tilde{u}}_{k-1}^{}$ into (A3), then:

(A7) $$\begin{array}{cc} {\tilde{u}}_{k}^{} & ={\tilde{u}}_{k-1}^{}-G_{k}H_{k}(\Phi_{k}F_{k-1}{\tilde{u}}_{k-1}^{}+\Gamma_{k}{\tilde{u}}_{k-1})-G_{k}H_{k}w_{k}-G_{k}\nu_{k}-G_{k}H_{k}\Phi_{k}\xi_{k-1}\\ & =(I_{p}-G_{k}H_{k}\Phi_{k}F_{k-1}-G_{k}H_{k}\Gamma_{k}){\tilde{u}}_{k-1}^{}-G_{k}H_{k}w_{k}-G_{k}\nu_{k}-G_{k}H_{k}\Phi_{k}\xi_{k-1}\\ & =(I_{p}-G_{k}D_{k}){\tilde{u}}_{k-1}^{}-G_{k}H_{k}w_{k}-G_{k}\nu_{k}-G_{k}H_{k}\Phi_{k}\xi_{k-1} \end{array}$$

Take the mathematical expectation at both sides of (A7), then:

(A8) $$E[{\tilde{u}}_{k}^{}]=(I-G_{k}D_{k})E[{\tilde{u}}_{k-1}^{}].$$

According to $G_{k}D_{k}=\Lambda_{k-1}^{}D_{k}^{T}\Psi_{k}D_{k}$ and $\Lambda_{k}^{}=\Lambda_{k-1}^{}-\Lambda_{k-1}^{}D_{k}^{T}\Psi_{k}D_{k}^{}\Lambda_{k-1}^{}$, the spectral norm of $\left(I_{p}-G_{k}D_{k}\right)$ is less than 1 for all time. Then, $E({\tilde{u}}_{k}^{})$ tends to zero when k→∞, and:

(A9) $$E[{\tilde{x}}_{k}]=E[\xi_{k}]+F_{k}E[{\tilde{u}}_{k}^{}]=0.$$

## Appendix B

According to Equation (10), then:

(A10) $$\Delta y_{k|k-1}≜y_{k}-y_{k-1}^{}=H_{k}x_{k}+v_{k}+v_{c}^{}-H_{k-1}x_{k-1}-v_{k-1}-v_{c}^{},$$

(A11) $$\Delta y_{{}_{k|k-1}}^{p}≜y_{k}^{p}-y_{k-1}^{p}=H_{k}(\Phi_{k}\Phi_{k-1}{\hat{x}}_{k-2}+\Phi_{k}\Gamma_{k-1}{\hat{u}}_{k-2}+\Gamma_{k}{\hat{u}}_{k-1})-H_{k-1}(\Phi_{k-1}{\hat{x}}_{k-2}+\Gamma_{k-1}{\hat{u}}_{k-2}),$$

where $v_{c}^{}$ represents the unknown sound error, $y_{k}^{p}=H_{k}{\hat{x}}_{k|k-2}^{}$ and $y_{k-1}^{p}=H_{k-1}{\hat{x}}_{k-1|k-2}^{}$. Then, we have:

(A12) $$\Delta y_{k|k-1}-\Delta y_{{}_{k|k-1}}^{p}=C_{k}x_{k}+v_{k}-H_{k-1}x_{k-1}-v_{k-1}-H_{k}(\Phi_{k}\Phi_{k-1}{\hat{x}}_{k-2}+\Phi_{k}\Gamma_{k-1}{\hat{u}}_{k-2}+\Gamma_{k}{\hat{u}}_{k-1})+H_{k-1}(\Phi_{k-1}{\hat{x}}_{k-2}+\Gamma_{k-1}{\hat{u}}_{k-2})$$

Suppose that the system is time-invariant for a short period of time, where $\Phi_{k}\approx \Phi_{k-1}\approx I$,$H_{k}\approx H_{k-1}$, and $\Gamma_{k}\approx \Gamma_{k-1}\approx I_{3}$. Then, we have:

(A13) $$\begin{array}{cc} \Delta y_{k|k-1}-\Delta y_{{}_{k|k-1}}^{p} & =C_{k}x_{k}+v_{k}-H_{k-1}x_{k-1}-v_{k-1}-H_{k}(\Phi_{k}\Phi_{k-1}{\hat{x}}_{k-2}+\Phi_{k}\Gamma_{k-1}{\hat{u}}_{k-2}+\Gamma_{k}{\hat{u}}_{k-1})+H_{k-1}(\Phi_{k-1}{\hat{x}}_{k-2}+\Gamma_{k-1}{\hat{u}}_{k-2})\\ & \approx H_{k}(\Phi_{k}\Phi_{k}x_{k-2}+\Phi_{k}\Gamma_{k}u_{k-2}+\Gamma_{k}u_{k-1}+\Phi_{k}w_{k-2}+w_{k-1}-\Phi_{k}\Phi_{k}{\hat{x}}_{k-2}-\Phi_{k}\Gamma_{k}{\hat{u}}_{k-2}-\Gamma_{k}{\hat{u}}_{k-1})\\ & -H_{k}(\Phi_{k}x_{k-2}+w_{k-2}+\Gamma_{k}u_{k-2}-\Phi_{k}{\hat{x}}_{k-2}-\Gamma_{k}{\hat{u}}_{k-2})+(v_{k}-v_{k-1})\\ & =H_{k}(\Phi_{k}(\Phi_{k}x_{k-2}-x_{k-2})+(\Phi_{k}w_{k-2}-w_{k-2})-(\Phi_{k}\Phi_{k}{\hat{x}}_{k-2}-\Phi_{k}{\hat{x}}_{k-2}))+H_{k}w_{k-1}+(v_{k}-v_{k-1})\\ & \approx H_{k}w_{k}+(v_{k}-v_{k-1}) \end{array}$$

Then, the covariance of observation noise can be calculated as follows:

(A14) $${\hat{R}}_{k}^{\mathrm{SOD}}\approx [Var((y_{k}-y_{k-1}^{})-(y_{k}^{p}-y_{k-1}^{p}))-H_{k}Q_{k-1}H_{k}^{T}]/2.$$
